# Sensitive and critical periods in visual sensory deprivation

**DOI:** 10.3389/fpsyg.2013.00664

**Published:** 2013-09-26

**Authors:** Patrice Voss

**Affiliations:** ^1^Cognitive Neuroscience Unit, Montreal Neurological Institute, McGill UniversityMontreal, QC, Canada; ^2^International Laboratory for Brain, Music and Sound ResearchMontreal, QC, Canada

**Keywords:** blindness, crossmodal plasticity, early and late blind, critical periods, sensitive periods

## Abstract

While the demonstration of crossmodal plasticity is well established in congenital and early blind individuals, great debate still surrounds whether those who acquire blindness later in life can also benefit from such compensatory changes. No proper consensus has been reached despite the fact that a proper understanding of the developmental time course of these changes, and whether their occurrence is limited to—or within—specific time windows, is crucial to our understanding of the crossmodal phenomena. An extensive review of the literature reveals that while the majority of investigations to date have examined the crossmodal plasticity available to late blind individuals in quantitative terms, recent findings rather suggest that this reorganization also likely changes qualitatively compared to what is observed in early blindness. This obviously could have significant repercussions not only for the training and rehabilitation of blind individuals, but for the development of appropriate neuroprostheses designed to aid and potentially restore vision. Important parallels will also be drawn with the current state of research on deafness, which is particularly relevant given in the development of successful neuroprostheses (e.g., cochlear implants) for providing auditory input to the central nervous system otherwise aurally deafferented. Lastly, this paper will address important inconsistencies across the literature concerning the definition of distinct blind groups based on the age of blindness onset, and propose several alternatives to using such a categorization.

The scientific literature has grown rich in research illustrating the remarkable ability of the brain to reorganize itself following sensory loss. In particular, visually deafferented regions within the occipital cortex of early blind individuals have been repeatedly shown to be functionally recruited to carry out a wide variety of non-visual tasks. While the demonstration of crossmodal plasticity is well established in congenitally (CB) and early blind (EB) individuals, significant debate surrounds whether those who become blind later in life can also benefit from such compensatory changes. For instance, several initial neuroimaging reports (e.g., Cohen et al., 1999; Sadato et al., 2002) suggested that the crossmodal plastic phenomena observed in the blind are likely regulated by a particular *critical period* beyond which no observable changes occur. However, a number of other studies (e.g., Büchel et al., 1998; Burton et al., 2002a,b; Voss et al., 2006) have demonstrated that such crossmodal plastic phenomena might instead be regulated by a *sensitive period*, as opposed to a more rigid critical period, where sensory experience has a relatively greater influence on behavioral and cortical development, but is not necessarily exclusive to that period. Consequently, work to date has focused on the amount of measurable crossmodal plasticity as a function of the age of blindness onset, thus more or less assuming that differences observed between groups of individuals with differing onsets are quantitative in nature (i.e., individuals with an earlier blindness onset will show more crossmodal recruitment of occipital cortex than those with a later onset). Nonetheless, in light of recent findings, one could argue that the plastic changes that occur following blindness do not only change quantitatively with increasing age of blindness onset, but also qualitatively in that crossmodal recruitment of occipital cortex might reflect different processes and purposes for EB and late blind (LB) individuals. For instance, the functional relevance of crossmodal plasticity observed in late-onset blindness has yet to be clearly established, whereas it has been clearly linked to behavior in EB; thus even if we were to observe similar levels of crossmodal recruitment of visual areas in both EB and LB, the observed occipital activations may not share the same functional or behavioral relevance for both blind groups. As a result, we should perhaps no longer simply investigate the presence or absence of plasticity in early and late-onset blindness, but more importantly ask ourselves how the plastic processes and mechanisms change with increasing age of onset. Evidence supporting this claim will be discussed in detail below, following a brief primer on some general plastic properties of the visual system and an in-depth review of findings depicting the crossmodal plasticity phenomenon observed in early blindness.

## Plasticity in the visual system

Much of what we know today on the brain and its plastic properties, we owe in great part to the pioneering work of Nobel laureates David H. Hubel and Torsten N. Wiesel performed in the early 1960s. Their investigations on the effects of monocular deprivation revealed both an early innate period of development and a later critical period of experience-dependant plasticity. Indeed, their choice to deprive young kittens of vision in only one eye allowed them to directly compare the responses of both eyes, thus acting as an internal control for variations in the developmental stage of the animal. They showed that monocularly depriving newborn kittens for a least a month induced a dramatic shift in the primary visual cortex (V1) responses from the deprived eye to the non-deprived eye (over 98% of the recorded neurons were unresponsive to input to the formerly deprived eye) (Wiesel and Hubel, 1963). Follow up studies revealed that when kittens were binocularly deprived from birth, more than half of the cells continued to respond to both eyes (Wiesel and Hubel, 1965), and that when eyes were kept from working together by alternating occlusion of the two eyes, nearly all of the cells stopped responding to both eyes and were instead driven by one eye or the other (Hubel and Wiesel, 1965). These findings led them to hypothesize that the loss of deprived-eye responses was a result of competitive processes with the non-deprived eye and not simply from disuse.

Of greater relevance to the current special topic, Hubel and Wiesel (1970) later investigated whether these physiological effects were governed by a period of susceptibility; that is when these effects were greatest and how long they lasted, the duration of deprivation necessary to produce a change, as well as the relationship between the timing of deprivation and the ability to recover normal function. To address these issues, they deprived kittens for various periods of time at different ages and compared neural responses in the striate cortex from both monocular inputs. Importantly, they first showed that a period of susceptibility did in fact exist, starting early in the 4th week following birth and remaining high for approximately three weeks, only to slowly decline until the end of the third month. What really highlights the importance of this period is the fact that a monocular deprivation occurring during the first three months—even one as short as 3 or 4 days—leads to a lasting and largely irreversible decline in the proportion of cells responding to the deprived eye, whereas very long periods of monocular deprivation in the adult cat has very little to no physiological effects (Hubel and Wiesel, 1970). This observation of a critical period of susceptibility to deprivation was among the first to reveal the high degree of sensitivity of the immature brain to an altered sensory state during a very restricted time period in life.

It is probably pertinent at this point to make an important distinction between two related concepts; that is the difference between a sensitive period and a critical period. While both concepts have at times been interchangeable to a certain extent in the literature, they are best segregated to explain distinct developmental phenomena. Sensitive periods generally refer to a limited time window in development during which the effects of experience on the brain are unusually strong, whereas a critical period is defined as a special class of sensitive periods where behaviors and their neural substrates do not develop normally if appropriate stimulation is not received during a restricted period of time (Knudsen, 2004). The above-mentioned studies on monocular deprivation are perfect examples of critical periods, where the absence of normal sensory input during a specific time window leads to irreversible changes in brain function and connectivity. Indeed, if normal binocular input is not achieved by three months of age in kittens, no cells will ever respond to input from the occluded eye, even if visual input to the occluded eye is restored after the critical period.

So far the focus has been on an animal model of monocular deprivation to illustrate the importance of time windows in development during which competitive processes determine the role played by individual cells in the primary visual cortex. An important question that has not been raised yet concerns what happens when no visual input reaches the visual processing centers of the brain (i.e., binocular deprivation). Does the lack of sensory experience lead to disuse-related atrophic processes within these regions? Or are their still competitive processes at play to gain control of occipital cortical regions despite the lack of visual input? Such questions have led to many investigations and revealed that the blind constitute excellent models for studying the plastic nature of the brain (Bavelier and Neville, 2002; Pascual-Leone et al., 2005). The following section will describe in detail what we currently know about the consequences of complete blindness in human adults, both in terms of brain and behavioral changes.

## Extreme circumstances: the case of complete blindness

### Functional and behavioral adaptations

We have a pretty good understanding of how the brain processes visual information and of the specific roles played by various regions throughout the visual system. However, until recently, we had very little knowledge concerning what happened to these regions when an individual was cut off from the visual world due to peripheral lesions of the visual system (e.g., damage to the lens, retina or optic nerve) and thus leading to complete blindness. Evidently, progress has rapidly increased with the advent of specialized neuroimaging tools that allowed for the *in-vivo* investigation of the brain. The first neuroimaging studies used positron emission tomography (PET) to study the glucose metabolism of the occipital cortex at rest in both EB—individuals that become blind during the first few years of life (see Box 1)—and sighted individuals (Wanet-Defalque et al., 1988; Veraart et al., 1990). It was shown that the glucose metabolism observed in occipital cortex of blind individuals was greater than that observed in blindfolded sighted subjects, but comparable to what was observed when the blindfold was removed. These initial observations obviously raised important questions on the functionality of the EB's visual cortex. Subsequently, Uhl et al. (1991, 1993) were among the first to show task-related activations in response to tactile stimulation within occipital cortex of EB, and shortly thereafter came a multitude of brain imaging studies showing that their occipital cortex could be crossmodally activated by a variety of tactile (Sadato et al., 1996; Büchel et al., 1998; Burton et al., 2002a) and auditory (Weeks et al., 2000; Arno et al., 2001; Burton et al., 2002b) tasks.

Box 1: **Congenital blindness**: refers to individuals that were born blind, and as a result, were never exposed to visual stimulation.

**Early blindness**: refers to cases of blindness that occurred during the first few years of life, generally prior to the age of 5. However, there are multiple exceptions, with some studies including subjects up to 14 years of age in what are defined as early blind groups. Also, early blind groups often include congenitally blind individuals, unless otherwise specifically stated. While early and congenitally blind individuals are often pooled together, more recent studies have started segregating them into separate groups, as even a few years of visual experience could strongly alter the functioning and the anatomy of visual structures.

**Late blindness**: generally refers to cases of blindness that began after puberty (typically >16 years of age) or in adulthood. Again, there are exceptions to this with some studies including individuals with ages of onset as low as 7 years of age.

The lack of consistency in defining blind groups across studies has had two major consequences. The first is the often omittance of individuals with intermediate onsets of blindness (e.g., between 5 and 16 years of age), which of course introduces a strong sampling bias when attempting to relate the age of onset of blindness to a behavioral or neuroanatomical measure. The second is the undesired overlap between defined groups from different studies, where a given individual would be considered as ‘early blind’ in one and as “late blind” in others.

**Figure 1:**
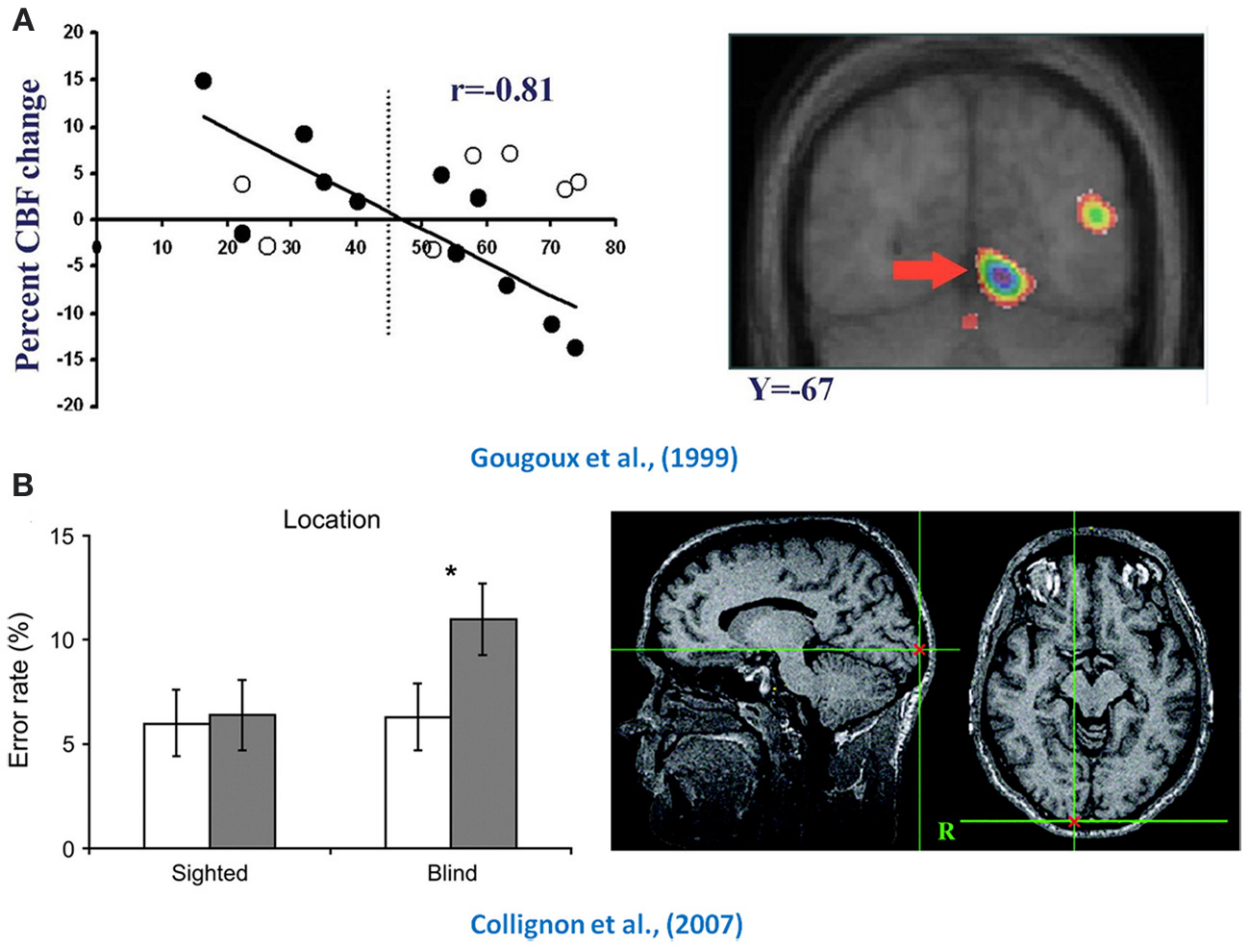
**Functional relevance of crossmodal plasticity**. Illustrated here are demonstrations of the functional role played by the occipital cortex in spatial hearing tasks in early blind individuals. The top row (panel **A**) depicts the finding that occipital activity in early blind individuals (black dots) was predictive of their performance in a sound localization task (Gougoux et al., 2005). The bottom row (panel **B**) illustrates the effect that TMS has when applied to the occipital cortex (black bars) when both blind and sighted subjects were asked to localize sounds (Collignon et al., 2007). Compared to Sham-TMS (white bars), TMS applied over occipital cortex reduced the performance of early blind subjects only, which is indicative that this region is functionally relevant for spatial processing in the early blind. Adapted with permission from Gougoux et al. (2005) and Collignon et al. (2007). ^*^P < 0.05.

Despite the impressive nature of the observed crossmodal activations in the occipital cortex, important questions still remained regarding their exact significance. Are they truly task-related or simply an epiphenomenon associated with the absence of visual input? Several findings suggest that the occipital cortex does indeed play a functional role in processing non-visual information following early blindness. The first line of evidence stems from research demonstrating strong correlations between brain activity in occipital cortex of EB and behavioral performance on a variety of tasks including verbal memory (Amedi et al., 2003), episodic retrieval (Raz et al., 2005) and sound localization (Gougoux et al., 2005). This is perhaps not so surprising given the wealth of evidence documenting the development of heightened compensatory perceptual and cognitive abilities in EB (see Voss et al., 2010). Auditory spatial abilities in particular have been heavily investigated in light of substantial questions concerning a blind person's ability to form adequate spatial representations in the absence of vision; consequently, an abundance of compelling evidence linking occipital functioning and sound localization in early blindness has been brought to light (see Figure 1; see also Collignon et al., 2009).

Additional evidence supporting the functional relevance of the crossmodal recruitment of occipital cortices in early blindness comes from the use of trans-magnetic stimulation (TMS) which enables inferences on causality via the temporary disruption of cortical functioning within very specific brain areas. Indeed, the application of TMS to occipital areas significantly hampers the performance of EB in tasks assessing sound localization (Collignon et al., 2007), verbal memory (Amedi et al., 2004) and Braille identification (Cohen et al., 1997), while leaving the performance scores of sighted individuals unaffected. Perhaps the most striking form of evidence comes from a blind expert Braille reader, who completely lost the ability to read Braille following an ischemic stroke causing bilateral lesions to her occipital cortex (Hamilton et al., 2000). Similarly, a middle-aged blind individual was reported as having transient difficulties in reading Braille while he experienced temporary visual hallucinations (Maeda et al., 2003). The fact that his ability returned to normal following the hallucinations suggests a causal relationship between occipital functioning and Braille reading in this blind individual. Taken together, these findings suggest that occipital cortex might still serve some functional purpose following blindness. What is not clear at this point, however, is how these crossmodal plastic adaptations come to be? Properly understanding how non-visual sensory inputs are processed within occipital cortex is a challenging task and is discussed in the following section.

### Crossmodal plasticity: underlying mechanisms

As highlighted earlier, many neural processes and connections are the result of competitive interactions between different neurons and sensory inputs, and as previously suggested by Pascual-Leone and Hamilton (2001), visual inputs might actually gain access to occipital regions by means of such competitive processes with the other senses during early development. One popular hypothesis is that occipital cortex might be by design best suited to carry out predetermined specialized functions for which the visual system provides the most adequate sensory input. However, in the case of blindness, other senses providing potentially relevant sensory input could gain access to the “visual” regions of the brain for further processing. Such a view therefore assumes that the functional specialization of “visual” cortical regions is preserved in blindness, and indeed there are a growing number of findings that support it.

For instance, regions specializing in the spatial processing of sounds in blind individuals appear to map onto areas of the dorsal visual stream known for similar processing of visual stimuli (Collignon et al., 2009, 2011). Another area well known for its functional specialization is the lateral-occipital complex (LOC), typically involved in object/form recognition processes, which has been shown on several occasions to be responsive to non-visual form processing in EB (Amedi et al., 2007, 2010). Similarly, the visual word form area, which, as its name indicates, responds well to the visual presentation of words, has been shown to be highly responsive to tactually presented Braille words in EB subjects (Reich et al., 2011). Furthermore, Pietrini et al. (2004) had previously shown that the tactile exploration of faces activated different regions than those elicited by the exploration of objects in the blind, suggesting that the development of topographically organized, category-related representations in extrastriate visual cortex does not require visual experience. Similarly, distinct regions within the ventral visual pathway of blind individuals show neural specialization for non-living and living stimuli in the auditory modality, suggesting that the conceptual domain organization in the ventral visual pathway does not require visual experience to develop (Mahon et al., 2009). Lastly, another well known area for its functional specialization is the human extrastriate cortical region known as the middle temporal complex (hMT+), which is highly responsive to visual motion. Several studies have shown that this region in blind individuals becomes responsive to both tactile motion on the fingers (Ricciardi et al., 2007) as well as to moving sound stimuli (Poirier et al., 2006). These findings, taken together, provide compelling evidence that the functional specialization of occipital regions is preserved in early blindness, and that the operations subserved by each region need not depend on visual input to be solicited by a given task.

Although many higher tier visual areas seem to have preserved there functional specialization following blindness, it is still undetermined how the non-visual input reaches occipital cortex. Two obvious possibilities are either via already existing connections or through the establishment of new connections not present in sighted individuals. The former could result from the unmasking or strengthening of latent pre-existing pathways between sensory-specific cortices and/or between multisensory areas and occipital cortex. The latter, however, appears unlikely for at least two reasons. The first, as discussed later on, stems from a growing body of evidence demonstrating that crossmodal recruitment of occipital cortex is possible in normal sighted individuals after brief transient periods of visual deprivation, which suggests that already existing intermodal connections are at play [see reviews on potential multisensory pathways by Schroeder et al. (2003); Cappe et al. (2009)]. The second, results from animal work investigating the developmental synaptic pruning period in early infancy. It has been shown that corticocortical projections from auditory to visual cortex are present in infant kittens only to be soon after pruned away due to competitive processes (Innocenti and Clarke, 1984; Innocenti et al., 1988). However, in kittens deprived of vision at birth, these extrinsic connections to the occipital cortex seem to remain (Berman, 1991; Yaka et al., 1999). These findings rather suggest that it is the strengthening of normally transient intermodal connections, and not the formation of new connections following blindness, that is likely to provide the substrate for the crossmodal innervation of occipital cortex following early blindness.

Research with animal models of blindness has illustrated several such pathways that could potentially mediate the crossmodal processing of sound in blindness. For instance, studies with blind rodents have shown the existence of connections between the inferior colliculus (an important auditory relay) and the lateral geniculate nucleus (LGN—an important visual relay) (Doron and Wollberg, 1994; Izraeli et al., 2002), suggesting that auditory information may reach the occipital cortex via the optic radiations ascending from the LGN. Alternatively, auditory information could be fed via direct connections between the medial geniculate nucleus (MGN—an important auditory relay) and the occipital cortex (Laemle et al., 2006). Furthermore, Karlen et al. (2006) have shown that the occipital cortex of CB oppossums receives projections from not only the auditory (MGN), but also from the somatosensory (ventral posterior) nucleus of the thalamus, thus suggesting a possible route for tactile information to be conveyed toward the occipital cortex. More recently, the findings of Laramée et al. (2011) suggest that corticocortical pathways could also mediate the crossmodal input into deafferented visual areas by showing indirect connections between the primary auditory and the primary visual cortex in visually deprived mice.

Anatomical tracer studies in normally seeing primates have shown the existence of direct connections going from caudal auditory areas to peripheral V1/V2 (Falchier et al., 2002; Rockland and Ojima, 2003), suggesting that the necessary pathways to mediate crossmodal plasticity likely exist prior to visual deprivation. Evidence in humans is a little sparser, but several recent findings also support corticocortical pathways between auditory and visual areas as a likely source for streaming auditory input into the occipital cortex. For instance, a recent diffusion tensor imaging (DTI) tractography study in normal seeing humans has revealed the existence of connections between Heschl's gyrus and the calcarine sulcus (Beer et al., 2011). Whether this pathway is different in blind individuals has yet to be established, although it perhaps need not be to subserve the crossmodal recruitment of visual areas by sound. Moreover, a pair of recent studies used dynamic causal modeling (DCM) to investigate the effective connectivity between regions underlying auditory activations in the primary visual cortex of EB individuals. DCM is a powerful hypothesis-driven tool that allows for inferences on the causality between the activity observed in different brain areas and, analogously, to study how information flows in the brain (Friston et al., 2003). It was found that auditory-driven activity in V1 is best explained by direct connections with A1 (Collignon et al., 2013) and that the connectivity between both structures was stronger in the blind compared to sighted individuals (Klinge et al., 2010). A final argument in favor of corticocortical pathways underlying auditory recruitment of occipital areas stems from neuroanatomical investigations showing the optic radiations (geniculocortical tracts) of EB humans to be severely atrophied (Noppeney et al., 2005; Shimony et al., 2006; Pan et al., 2007; Park et al., 2007; Ptito et al., 2008), rendering them unlikely candidates for relaying auditory information to visually deafferented cortical areas.

## Crossmodal plasticity in blindness: bounded by critical or sensitive periods?

So far only research findings relating to early or congenital blindness have been covered (see Box 1), more or less ignoring the notion of critical periods. This is partly due to the fact that most research has primarily focused on the effects of early blindness, and also because, there is little consensus on the effects of late-onset visual deprivation. The following sections attempt to disentangle the different findings relating to late blindness and to contrast them with those relating to early blindness.

One of the first neuroimaging studies to investigate the occipital brain metabolism in EB individuals (Veraart et al., 1990) also examined a group of LB individuals. It was shown that occipital functioning in LB was different from that of EB: while EB were found to have higher occipital glucose metabolism relative to sighted individuals, LB showed a reduction. This finding obviously served as an early indication that the age of blindness onset was potentially a determining factor in the changes that occur in occipital cortex following visual deprivation. Indeed, a pair of early investigations of task-related activations showed that while crossmodal recruitment was observed in EB, no such observation was made in LB (Cohen et al., 1999; Sadato et al., 2002). This finding suggested the existence of a strict critical period for the development of crossmodal plasticity within the occipital cortex (14 years of age: Cohen et al., 1999; 16 years of age: Sadato et al., 2002), after which no crossmodal reorganization would take place if the onset of blindness occurred beyond this period. However, findings from a large number of other studies have since challenged this view. Kujala et al. (1997) first suggested the possibility of crossmodal reorganization in LB individuals by showing posterior event-related potential (ERP) responses similar to those observed in EB when they performed sound-change detection tasks. Subsequently, a PET study revealed activation of visual cortex, albeit manifesting somewhat different patterns, during Braille reading and auditory word processing in both EB and LB subjects (Büchel et al., 1998). This was later followed by a series of studies by Burton et al. in which LB were shown to activate occipital regions in response to a variety of tactile and auditory tasks (Burton et al., 2002a,b, 2003, 2004, 2006; Burton and McLaren, 2006). Similarly, several auditory spatial tasks elicited occipital activations in late-onset blind individuals (Voss et al., 2006, 2008, 2010). However, these crossmodal changes were not accompanied by behavioral enhancements, as is the case in EB individuals, raising questions concerning the functional relevance of the observed crossmodal plasticity in LB.

Despite some exceptions, there thus appears to be some agreement that crossmodal recruitment of deafferented visual areas is not exclusive to EB and can be observed in cases of late-onset blindness as well. While this is the case, the crossmodal recruitment in LB appears to be nonetheless generally reduced (both in terms of intensity and spatial extent) relative to EB, suggesting that while the development of crossmodal plastic processes might not be bound by a critical period, it is definitely modulated by a sensitive period in early development during which reorganization is likely to be more pronounced.

### Crossmodal changes in sighted individuals

Additional evidence supporting the existence of adult crossmodal plasticity stems from research investigating the effects of temporary visual deprivation in normal sighted individuals. One of the first studies to document such effects revealed that short-term light deprivation enhances the excitability of visual cortex. Indeed, a brief period of visual deprivation was shown to not only induce a reduction in the TMS thresholds required for eliciting phosphenes but also lead to an increase in visual cortex activation by photic stimulation (Boroojerdi et al., 2000). Subsequently, using a pharmacological approach in combination with TMS, it was shown that GABA, NMDA, and cholinergic receptors likely play an important role in rapid experience-dependent plasticity in visual cortex, as administering appropriate agonists/antagonists eliminated the TMS phosphene-threshold decrease associated to transient visual deprivation (Boroojerdi et al., 2001).

These findings were soon followed by research inspired by a school for the blind in Spain, which required that its instructors experience daily life without sight for an entire week during training (Pascual-Leone and Hamilton, 2001). The instructors reported having heightened awareness for sounds, being able to better distinguish different speakers and to better orient themselves in response to incoming sounds. To follow up on these reports, Pascual-Leone and Hamilton (2001) developed a protocol in which sighted volunteers would be blindfolded for 5 days. Preliminary findings revealed an increase in BOLD signal within the occipital cortex in response to tactile stimulation after 5 days of complete visual deprivation, and that this increase was no longer present the day following blindfold removal. These findings indicated that rapid crossmodal changes can occur in the occipital cortex of adults when temporarily deprived of vision, and were further documented in Merabet et al. (2008). Remarkably, such crossmodal deprivation-related effects were limited to the blindfolding period and were rapidly reversible.

**Figure 2:**
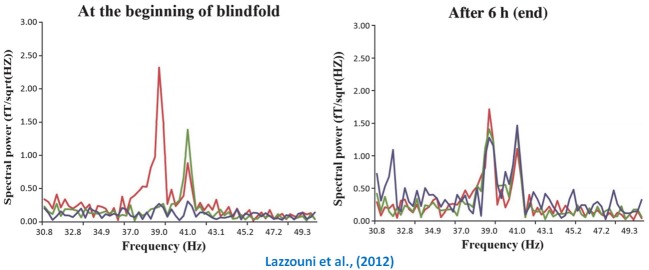
**Crossmodal plasticity in temporarily deprived sighted individuals**. This figure portrays a recent MEG finding that testifies to the impressive speed at which the visual cortex can display auditory cortex-like functioning following a short period of visual deprivation. The left graph shows that prior to blindfolding the two spectral peaks (left temporal in red; right temporal in green) associated with modulation rate of the auditory stimuli presented to both ears (39 and 41 Hz) are clearly restricted to the temporal electrodes (auditory cortex). However, as shown in the right graph, the same peaks can now be found in visual cortex (purple peaks) following a 6 h visual deprivation period. Adapted with permission from Lazzouni et al. (2012).

Subsequent work has impressively shown that very short time periods of visual deprivation are sufficient to induce marked crossmodal changes in occipital cortex. For instance, Weisser et al. (2005) demonstrated that 2 h of visual deprivation was enough to induce the neural changes for the processing of tactile shapes within the occipital cortex of normally sighted individuals. In a recent study, we used a novel technique to determine whether occipital cortex processes auditory input in a similar manner to auditory cortex (Lazzouni et al., 2012). We developed a blindfolding protocol to assess the effects of short-term visual deprivation on the auditory steady state response (ASSR). The ASSR can be defined as an electrophysiological response to rapidly changing auditory stimuli, where neuronal populations respond at the same frequency as the modulation rate of an amplitude-modulated (AM) tone and, importantly, for which the sources of the activity can be extracted using dipole analyses. The ASSR therefore constitutes a powerful tool as it evokes a response that is intrinsically linked to the stimulus and can be tracked within the brain. The results showed that the two spectral peaks associated with the modulation rates of two dichotically presented stimuli (39 and 41 Hz) were observed only within auditory cortex prior to blindfolding. Following 6 h of visual deprivation, however, two peaks were also observed in occipital cortex (see Figure 2), thus shedding light on the timeline associated with short-term crossmodal recruitment of input-deprived sensory cortices. This finding also demonstrates that visual cortex can display auditory cortex-like functioning in response to auditory input during periods of deprivation.

### Crossmodal plasticity: early- vs. late-onset blindness

**Figure 3:**
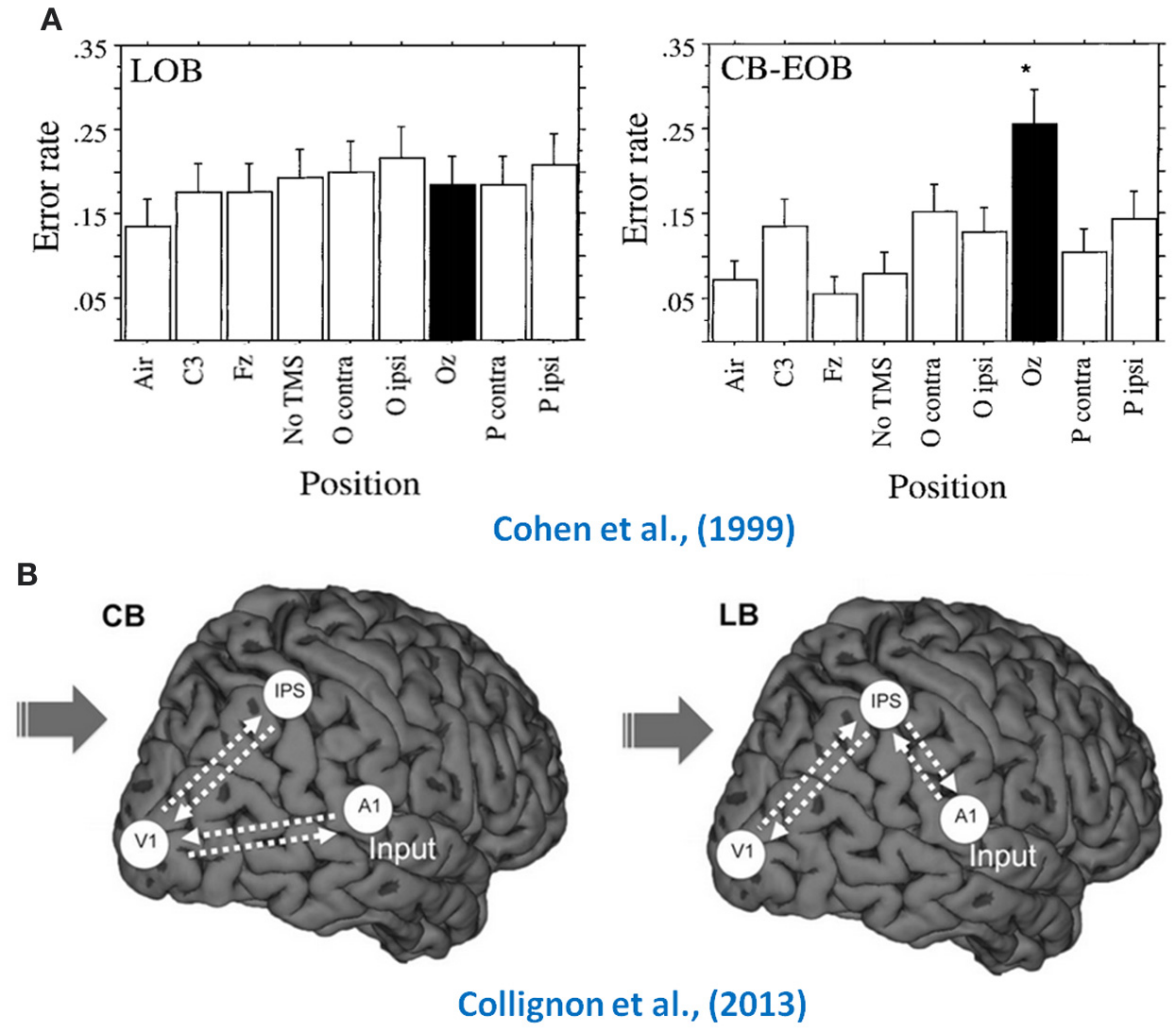
**How early and late blind differ**. Illustrated here are two examples of how the crossmodal plasticity observed in early and late blind individuals differs. The top row (panel **A**) illustrates the differential effect TMS has when applied over the occipital cortex (black bars) of LB (first bar graph) and EB (second bar graph) on their performance in a Braille task, where only the early blind showed an increase in error rate (Cohen et al., 1999). The bottom row (panel **B**) consists in a schematic representation of how auditory information flows toward V1 in the congenitally blind and late blind, illustrating the DCM findings of Collignon et al. (2013). Adapted with permission from Cohen et al. (1999) and Collignon et al. (2013). ^*^p < 0.001.

The previous sections documented multiple demonstrations of the crossmodal processing that occurs in the mature occipital cortex. However, an important question to ask concerns whether the plasticity observed in the adult brain is similar to what is observed in the visually deprived immature brain. Aside from the typical observation of reduced crossmodal recruitment in LB (with the exception of Büchel et al. (1998) who reported greater activation in LB), the following sections will highlight four major distinctions between the crossmodal changes observed for early and late onset blindness that argue for the existence of important underlying functional differences between the two (see also Figure 3). Indeed these findings point not only to quantitative differences (i.e., the amount of crossmodal recruitment observed) between the compensatory reorganization that occurs following early and late onset blindness, but also to qualitative ones relating to, for instance, the underlying mechanisms of crossmodal recruitment and its functional relevance to behavior.

#### Functional relevance of crossmodal processing

As highlighted above, there is an abundance of evidence demonstrating the functional relevance of the crossmodal recruitment of occipital areas in EB. Several studies have showed strong correlations between behavioral performance and occipital activity (Amedi et al., 2003; Gougoux et al., 2005; Raz et al., 2005), whereas others have shown that the temporary (Cohen et al., 1997; Amedi et al., 2004; Collignon et al., 2007) and permanent (Hamilton et al., 2000) dysfunction of occipital neurons interferes with performance in non-visual tasks. Interestingly, there is little to no evidence of this in LB. This is likely in part due to the limited evidence of enhanced perceptual abilities in LB, as they are often found to be indistinguishable from sighted individuals in terms of performance. The observed crossmodal recruitment in LB therefore seemingly doesn't lead to any behavioral gain as it does in the EB. This assumption is supported by data provided by Cohen et al. (1999), where performance on a Braille reading task was unaffected in LB by the application of TMS over occipital cortex, whereas it reduced performance in EB. While there are a few exceptions where LB have demonstrated heightened perceptual abilities compared to sighted individuals (e.g., Voss et al., 2004), such instances have generally not been associated with increased crossmodal plasticity. Indeed, several other factors could explain increased performance (e.g., training, experience) without the involvement of occipital regions.

One previously proposed hypothesis to explain occipital activations observed in the late-blind stated that they might be the result of mental imagery processes. It was reported by Büchel et al. (1998) that their LB subjects immediately transformed tactile and auditory cues into a visual representation, implying that any occipital activation could be due to “visualization” of the task. While such visual imagery processes have been shown to activate components of the visual system in normal sighted individuals (Kosslyn et al., 1995), more recent paradigms, however, have shown that occipital recruitment necessitates more active tasks that explicitly require subjects to use visual imagery (Kosslyn et al., 2001). Moreover, the visual imagery hypothesis loses traction when considering that occipital recruitment is seldom observed in the sighted when performing non-visual tasks that are also performed by the blind. This would imply that the unlikely scenario where LB resort to visual imagery and not sighted individuals takes place. In fact, it is often reported that when sighted individuals perform non-visual tasks, cross-modal inhibitory mechanisms are engaged (e.g., occipital deactivation is observed) to reduce the functioning of cortices subserving the unattended (and potentially distracting) visual modality (e.g., Laurienti et al., 2002; Gougoux et al., 2005).

#### Attentional mechanisms/processes

One exception that has linked superior performance in LB to brain changes has done so using an auditory spatial change-detection task and ERP measurements (Fieger et al., 2006). LB participants were significantly more accurate than sighted participants at localizing/detecting deviant auditory stimuli in peripheral auditory space (performance for both groups was identical for central auditory positions). This was also a task for which the CB had been shown previously to excel at (Röder et al., 1999), and important differences were observed when comparing the ERP results from both studies. The N1 ERP component displayed a more sharply tuned spatial gradient during peripheral attention in CB than in the sighted group, whereas the P3 component was identical in both groups (Röder et al., 1999). Conversely, the early N1 amplitude to peripheral standard stimuli displayed no significant spatial tuning in either the LB or the sighted controls, whereas the amplitude of the later P3 elicited by targets/deviants displayed a more sharply tuned spatial gradient during peripheral attention in LB compared to controls (Fieger et al., 2006). As such, it appears that CB persons possess a more sharply tuned early attentional filtering, manifested in the N1 component, while LB show superiority at deploying late attentional processes of target discrimination and recognition, indexed by the P3 component. These findings therefore strongly suggest that even when both CB and LB individuals show a behavioral advantage over sighted subjects on a given task, these enhancements are potentially mediated by different underlying cerebral mechanisms.

#### Source of auditory input into the occipital cortex

The potential role played by corticocortical connections in mediating the crossmodal recruitment of occipital cortex was specifically underlined in previous sections. For instance, a DTI tractography analysis has shown the existence of direct connections between primary auditory and visual areas in normal seeing individuals (Beer et al., 2011), whereas the use of DCM enabled researchers to establish that the functional connectivity between both structures is stronger in EB than in sighted individuals (Klinge et al., 2010). To addresses the possible differences between EB and LB individuals, we have recently shown that the flow of auditory information into the occipital cortex might be mediated by a different pathway in LB using DCM analyses (Collignon et al., 2013). Since it was recently demonstrated, using DCM, that crossmodal plasticity observed in CB individuals is more likely to be supported by corticocortical connections rather than thalamocortical connections (Klinge et al., 2010), we included only corticocortical connections in our models. Our findings indicated that the auditory activity observed in occipital cortex of CB individuals was best explained by direct feed-forward connections from primary auditory to primary visual cortex, whereas in LB, auditory information appears to rely more on an indirect feedback route using parietal regions as a relay between both primary sensory areas (Collignon et al., 2013). This strongly suggests that the crossmodal recruitment of visually deafferented areas is likely mediated by different pathways in EB and LB.

Indeed, it is highly likely that EB individuals have access to different pathways given the excessive connectivity between regions in early development. Indeed, the synaptic density of visual cortex reaches levels greater than that of adults in early infancy through synaptogenetic processes, and then gradually decreases to adult levels by approximately 5 years of age through the pruning of exuberant connections (Johnson, 1997), a process that is interrupted by visual deprivation (Stryker and Harris, 1986). Moreover, as highlighted above, the existence of such corticocortical connections between auditory and visual areas has been shown in young infant animals, only for the majority of these connections to be pruned away during normal development (Innocenti and Clarke, 1984; Innocenti et al., 1988). Whether similar corticocortical connections are also more prominent during early development in humans is unclear, but if present, EB may utilize and strengthen these normally transient exuberant connections to compensate for the loss of sight through experience-dependent stabilization processes, whereas LB must rely on connections that develop within the normal visual brain.

#### Preserved functional specialization

More recent research has begun to examine whether the crossmodal takeover of occipital cortex due to blindness follows some sort of organizational principle. There are now several lines of evidence stemming from neuroimaging studies [reviewed in Voss and Zatorre (2012)] that illustrate how the pre-existing functional specialization of specific cortical regions appears to be preserved following visual deprivation. As discussed earlier, a well documented example of this concerns the LOC, notably involved in object/form recognition processes. Amedi et al. (2007, 2010) have shown on multiple occasions that this region is also recruited by auditory and tactile form recognition tasks in EB individuals. Similarly, the visual motion processing center (area MT) has been shown to be recruited by both tactile (Ricciardi et al., 2007) and auditory (Poirier et al., 2006) motion stimuli. Both of these examples convincingly suggest that visual deprivation does not alter the specialized modular organization of the visually deafferented occipital areas of the brain, and that the operations subserved by each region need not depend on visual input to be solicited by a given task. Importantly, with respect to the objectives of this paper, two recent investigations have comparatively investigated this topic in both CB and LB individuals. First, we have recently shown that while both recruit occipital regions for sound processing, the preferential activation of the right dorsal stream for the spatial processing of sounds (compared to spectral processing of sounds) was only observed in CB (Collignon et al., 2013). This suggests that these occipital regions maintain a functional specialization for spatial processing in other senses only if vision is lost early in life. A second example supporting such a claim was provided by Bedny et al. (2012) who investigated the role of the visual cortex in language processing in both CB and LB individuals. Again, while they observed that occipital cortex was recruited by general auditory input in both groups, a preferential response to speech stimuli in the left hemisphere (compared to non-speech) was only observed in CB, suggesting that early visual experience might be detrimental to the occipital cortex acquiring a role in language processing following blindness.

The above-mentioned points raise interesting questions concerning the role played by sensitive/critical periods. While the general observation of crossmodal recruitment in LB individuals suggests that it is subject to the influence of a sensitive period, the highlighted differences indicate that different processes might be mediating the observed crossmodal recruitment in both early and LB individuals. If this is indeed the case, it rather suggests that *critical periods* may play a role after all, with perhaps varying cutoff points with regards to the different processes in play. Consequently, future work would benefit from attempting to target these issues by relating the age of blindness onset with the development of specific particularities that so far have only been observed in early blindness (e.g., functional relevance of recruitment, corticocortical connectivity).

## Implications for sight restoration

What happens to the ability of the “visual” brain to process visual information once it “goes auditory?” Such a question has important repercussions when considering the potential outcomes of sight restoration procedures and prostheses. Over three centuries ago, the Irish philosopher William Molyneux posed an analogous question to one of his contemporaries, John Locke, on how long term blindness would affect one's ability to see should sight be restored (Degenaar, 1996): *“Suppose a man born blind, and now adult, and then taught by his touch to distinguish between a cube and a sphere of the same metal, and the same bigness, so as to tell, when he felt one and the other, which is the cube, which is the sphere. Suppose then, the cube and the sphere placed on a table, and the blind man to be made to see. Query, whether by sight, before he touched them, he could distinguish, and tell, which is the globe, which is the cube?”* While this matter has since been debated for decades on end between various historical figures, there have been several case studies that have provided some insight into the matter, demonstrating for instance that visual acuity is severely reduced after cataract removal surgery following prolonged periods of deprivation (von Senden, 1960; Gregory and Wallace, 1963; Fine et al., 2003). Additionally, recent neuroimaging data allows for the investigation of potential underlying mechanisms. As already noted, the visual brain goes through drastic changes that might significantly alter an individuals' ability to process visual information should sight be restored. The next section will, however, first examine research with deaf individuals, as technological advances for restoring hearing in profoundly deaf individuals have achieved a fair deal of success with the development of sophisticated cochlear implants (CI). Such progress has allowed researchers to ascertain the consequences of crossmodal plasticity in the deaf population on the success rate of CIs, and will therefore provide insight into how to approach the same issues in blindness.

### Insights from the deaf

Once they have become responsive to a new input modality, can the auditory cortices still process to their original source of input? This question bears special importance given that profound deafness can sometimes be reversed by auditory stimulation via a cochlear implant (CI) (Ponton et al., 1996). Put simply, the device replaces normal cochlear function by converting auditory signals into electrical impulses delivered to the auditory nerve (see Mens, 2007 for further details). Several studies have shown the existence of a critical period that cannot be exceeded for recovery of auditory functions following aural deprivation (Kral et al., 2005; Sharma et al., 2005). This time window is generally limited to the first few years of life, with evidence suggesting that if implanted before the age of 2, children can acquire spoken language in a comparable time-frame to normal hearing children (Waltzman and Cohen, 1998; Hammes et al., 2002).

Although it was initially thought that the duration of auditory deprivation should account for most of the variance of the implantation outcome, several lines of evidence clearly suggest other modulating factors (O'Donoghue et al., 2000; Lee et al., 2001; Sarant et al., 2001). In fact, a retrospective case review showed that the duration of deprivation only accounted for 9% of the variability in implant outcome (Green et al., 2007). An alternate predictor can be found, for instance, in preoperative measures of cerebral metabolism. Lee et al. (2001) for instance, showed that the temporal cortex becomes hypometabolic following auditory deprivation, and that the level of hypometabolism is correlated to speech comprehension scores obtained post-implantation. In other words, the longer a person has been deaf, the less likely it is that their temporal cortex will be hypometabolic and the more likely their speech perception capacity will be compromised. In the same vein, it was later shown that speech perception performance was negatively associated with activity in occipito-temporal networks (Lee et al., 2005), even when factoring out the confounding effect of age of implantation (Lee et al., 2007). Furthermore, other important processes may be also at play, such as the level of crossmodal reorganization of the auditory cortex (see Giraud and Lee, 2007). For instance, one study compared cortical evoked potentials involved in the processing of visual stimuli in implanted subjects (Doucet et al., 2006). After evaluating the speech perception abilities of the implanted subjects, they were subsequently divided into two groups based on their performance. It turned out that the group with the poorest performers for speech perception was also the one where implanted individuals showed broader and more anterior scalp distributions when processing visual stimuli (i.e., likely the result of crossmodal processing of the visual stimuli in temporal auditory areas), and vice-versa. It thus appears that several interacting factors influence the outcome of cochlear implantation, of which importantly is crossmodal reorganization. Awareness of this important fact will evidently have an important impact on how similar concerns will be addressed in blindness.

### Is the visual system still visual following blindness?

Knowing whether crossmodal plastic changes are reversible is crucial to the proper development of neuroprostheses designed to restore vision in blind individuals. Although significant progress has been made toward achieving such a goal, future research is extremely dependant on our understanding of how blindness affects the brain, and on how these effects are driven or modulated by the age of blindness onset. Indeed, the brain of a LB individual may be more apt to process visual input following a prolonged period of visual deprivation, whereas the brain of an EB individual has likely underwent permanent plastic changes rendering it unable to process visual information. For instance, the finding that the optic tracts and radiations are atrophied in EB (Noppeney et al., 2005; Shimony et al., 2006; Pan et al., 2007; Park et al., 2007; Ptito et al., 2008) raises serious questions about the integrity of the pathways and whether or not they could convey electrical information stemming from retinal, subretinal, or epiretinal implants (see Merabet et al., 2005), or even transmit retinal images obtained following cataract removal in individuals with congenital cataracts. Furthermore, the numerous reports of significant reduction of cortical gray matter in occipital cortex raises serious questions regarding the area's ability to process visual input (Pan et al., 2007; Ptito et al., 2008; Lepore et al., 2010). A compensatory approach more likely to provide a successful outcome in EB is the use of sensory-substitution devices, where one sensory modality is used to supply information normally gathered by the deprived sense. Perhaps the most well known example of this is Braille, which of course has been highly successful in providing information normally acquired through vision (e.g., reading material) via the tactile modality. Several more sophisticated devices -that transform visual information captured via cameras into spatially relevant tactile or auditory stimulation- have since been implemented (Meijer, 1992; Bach-y-Rita et al., 1998; Capelle et al., 1998) and have allowed blind individuals “to see” complex two-dimensional objects and shapes (e.g., Arno et al., 1999; Renier and De Volder, 2010), and more recently to even navigate around obstacles in a highly controlled environment (Chebat et al., 2011). While these devices are not at a point where they can be relied upon to successfully navigate in the real world, they provide nonetheless a very promising avenue for future research designed to aid visually deprived individuals.

Visual restoration, however, might still be possible for LB individuals. For instance, Pan et al. (2007) showed that white matter (WM) loss in the optic tract and radiation of EB individuals was modulated by the age of blindness onset, suggesting that a later onset would have less effect on the anatomical integrity of the visual pathways. Moreover, Schoth et al. (2006) found no evidence of WM loss in either visual cortex or in visual tracts in subjects that could be categorized as LB (with a mean age of blindness onset of twelve), suggesting that the visual pathways may still be able to communicate signals toward occipital cortex. Consequently, approaches that involve cataract removal and retinal implants are likely to be considerably more viable in individuals that benefitted from the normal development of the visual system.

## Future considerations

Given the influence early development has on the emergence of crossmodal plastic phenomena in blind individuals, what steps need to be taken to further our understanding of the different processes at play? A crucial first step will be to address inconsistencies across the literature regarding how blind individuals are segregated into different groups based on their age at the onset of blindness (e.g., EB and LB individuals). This segregation is often done in a very arbitrary manner, as very few studies use the same definitions to classify and circumscribe early and late onset blind groups, and in fact occasionally overlap across published reports. This is of course quite troublesome when wanting to compare findings across studies, and will require greater care and cooperation between research groups in order for future work to yield fruitful results.

As first highlighted in Box 1, the current lack of uniformity across studies in defining the range of onsets of blindness for EB and LB groups has yielded at least two substantial issues. The first relates to the often non-inclusion of a large group of blind individuals with ages of blindness onsets that lie between chosen cut-offs for both for early and late onset blind groups. This practice not only introduces a strong sampling bias, but also removes potentially important data when investigating blindness-induced crossmodal plasticity. Indeed, important developmental sensitive periods may take place during this gap in the ages of onset. The addition of one or more distinct blind groups covering this gap could help alleviate the loss of potentially important information. In this vein, Li et al. (2013) very recently addressed this issue by defining four distinct groups in investigating brain anatomical connectivity networks: CB, EB (onset after birth but prior to the age of 12), adolescent blind (onset between 12 and 15 years of age inclusively) and LB (onset after 15 years of age). While the chosen ranges could be debated, this nonetheless represents an important first step in directing future research. This work also highlights the fact that it might also be wise to divide CB and EB individuals into separate groups (which several groups have started doing), as even a few years of visual experience could have a significant impact on the functional architecture of the visual system and on the manner it is crossmodally recruited following blindness. Indeed the use of a continuum of onsets of blindness will better allow for the direct investigation of the developmental time-course of processes that govern the emergence of crossmodal plasticity.

The second, potentially more serious issue arising from the inconsistent definitions, concerns the often overlapping of groups across different studies; i.e., a given blind individual could be categorized as an EB individual in one study and as a LB individual. For instance, Burton et al. (2002b, 2003, 2004, 2006) have often considered individuals with onsets of blindness occurring after the age of 7 as a LB individual; so has Fieger et al. (2006) and Bedny et al. (2012) for individuals with onsets occurring after the age of the 9. This is in stark contrast with other reports that have considered individuals with an onset occurring prior to 13 years of age as EB (e.g., Cohen et al., 1999; Sadato et al., 2002; Voss et al., 2008). This is a clear indication that greater effort and care should be put into homogenizing blind group definitions in order to better understand the effects sensitive and critical periods in sensory deprivation.

Lastly, the above-mentioned concerns could also be significantly alleviated by simply moving away from creating groups altogether. Certainly, it could be argued that we should be looking to use the age of blindness onset more as a continuous variable and search for non-linearities in the resulting functions linking the age of onset with various dependant variables, which would be indicative of sudden changes in the occurrence of crossmodal plasticity and possibly resulting from important critical or sensitive periods. For instance, if crossmodal plasticity changes only quantitatively over time, than the relationship between the age of blindness onset and various dependant variables would be a linear one. However, as discussed above, there are several lines of work suggesting that the crossmodal plastic process also undergoes some qualitative changes with later onsets, suggesting that the relationship could in fact be non-linear. Such an approach would have multiple benefits, perhaps none greater than the removal of the group definitions which are often highly arbitrary and the cause of discrepancies between studies. Moreover, treating the age of blindness onset as a continuous variable should allow for the extraction of important time-points during the development of crossmodal plastic phenomena in a data-driven way, rather than by the use of a-priori definitions of particular subgroups based on the age at blindness onset.

### Conflict of interest statement

The author declares that the research was conducted in the absence of any commercial or financial relationships that could be construed as a potential conflict of interest.
